# Exposure to liquid sweetness in early childhood: artificially‐sweetened and sugar‐sweetened beverage consumption at 4–5 years and risk of overweight and obesity at 7–8 years

**DOI:** 10.1111/ijpo.12284

**Published:** 2018-04-06

**Authors:** A. K. Macintyre, L. Marryat, S. Chambers

**Affiliations:** ^1^ Centre for Health Policy University of Strathclyde Glasgow UK; ^2^ Farr Institute Scottish Collaboration for Public Health Research and Policy University of Edinburgh Edinburgh UK; ^3^ MRC/CSO Social and Public Health Sciences Unit University of Glasgow Glasgow UK

**Keywords:** Artificially sweetened beverages, longitudinal cohort, obesity, sugar‐sweetened beverages

## Abstract

**Background:**

A significant gap exists in longitudinal evidence on early exposure to artificially sweetened beverages (ASBs) and weight outcomes for paediatric populations.

**Objective:**

The objective of this study is to examine the relationship between ASB/sugar‐sweetened beverage (SSB) consumption at 4–5 years and risk of overweight and obesity at 7–8 years.

**Methods:**

Data from a nationally representative cohort (n = 2986) in Scotland were analysed using logistic regression to evaluate the association between exposure to ASBs/SSBs at 4–5 years and risk of overweight and obesity at 7–8 years.

**Results:**

There were positive unadjusted associations between ASB consumption and risk of obesity, and following adjustment for confounders, ASB associations attenuated, and only the middle consumption category (1 to 6 times per week) remained significant (odds ratio 1.57, 95% confidence interval {CI} 1.05–2.36). For SSB consumption, there were no significant unadjusted associations, and following adjustment for confounders, only the middle consumption category was significant (odds ratio 1.65, 95% CI 1.12–2.44). There were no significant associations for risk of overweight.

**Conclusions:**

Longitudinal analysis from 4–5 to 7–8 years demonstrated some evidence of associations between ASBs/SSB consumption and risk of obesity. However, non‐linear patterns and wide CIs suggest cautious interpretation and need for future studies with long‐term follow‐up.

**Abbreviations**
ASBs: artificially sweetened beverages
BMI: body mass index
GUS: Growing Up in Scotland
SSBs: sugar‐sweetened beverages

## Introduction

Excess weight in childhood is a pervasive public health issue. A substantial proportion of children who are obese remains so in adulthood, leading to a range of chronic health problems 1. Highly processed sugary foods are widely available, and their high calorific value has been linked to excess energy intake and weight gain 2. The World Health Organization recommends that sugar intake in children and adults should be reduced to 5% of energy intake 3. Sugar‐sweetened beverages (SSBs) contribute to over 20% of UK children's added sugar consumption 4. The majority of systematic reviews demonstrate positive associations between increased SSB consumption and increased weight in children and adolescents, whilst a smaller number of reviews have found no or inconclusive evidence 5. Even with methodological issues 6, and concerns over industry funding 7, available evidence has formed the basis for levies on SSBs, such as in Mexico 2, and the forthcoming ‘sugar tax’ in the United Kingdom.

Encouragingly, there is evidence of declining SSB consumption in preschoolers in the United States 8. However, purchases of products containing artificial sweeteners and *both* caloric and artificial sweeteners have increased 2, 9. Indeed, artificially sweetened beverages (ASBs) have been promoted as an alternative to SSBs, with reformulation to decrease added sugars and increase non‐nutritive sweeteners a strategy to reduce the duty under taxation 10. However, concerns have been raised over ASBs as a weight management method 2, 11, 12. It is hypothesized that ASB consumption does not activate similar reward responses to sugar, and therefore, additional calories may be consumed to fulfil sweet cravings 11, 12.

Evidence on ASBs and weight gain is less conclusive than that of SSBs 2, 13, 14. Positive associations between ASB consumption and weight might result from individuals with higher weight being more likely to consume ASBs as a strategy to control weight 14. Two areas where evidence is particularly lacking for ASBs are (i) on long‐term outcomes associated with ASB consumption and (ii) the impact of ASB exposure for children 10, particularly in early childhood 13. Newby et al. 15 provide a rare investigation of exposure to ASBs in preschool children. Using data collected through a nutrition programme with low‐income families, they prospectively examined associations between beverage intakes of 2‐ to 5‐year‐olds and weight gain, measured at an interval of 6–12 months between two clinic appointments. No association was found for beverage intakes (including ASB consumption) and weight changes during this short follow‐up.

This study addresses this important evidence gap by examining ASB consumption at 4–5 years and risk of overweight and obesity at 7–8 years in a nationally representative cohort study from Scotland.

## Methods

### Study design

Growing Up in Scotland (GUS) is a national longitudinal study of three cohorts of children in Scotland 16. The study is funded by the Scottish Government and collects data on social, emotional and cognitive development, physical health and mental well‐being, family and community circumstances, employment, childcare and educational experiences 16. The nationally representative sample was achieved using a random sample of aggregated Data Zones, stratified by Local Authority Area and by Scottish Index of Multiple Deprivation. Data from birth cohort 1 were analysed here. Interviews were initiated in 2005/2006 when the children were 10 months old 16 (with the child's mother wherever possible), and follow‐up ‘sweeps’ were conducted over 10 years. At the first sweep, 5217 children were recruited. For this study, sweeps 1 (10 months), 4 (3–4 years), 5 (4–5 years), 6 (5–6 years) and 7 (7–8 years) were analysed. Data collection at each sweep was intended 6 weeks before the child's next birthday, and so exposure data were collected when most children were just under 5 and outcome data when they were just under 8 16.

### Eligibility criteria and exclusions

The Department of Work and Pensions used Child Benefit records to identify all children within each sampling unit who were eligible to participate, i.e. their date of birth fell between 1 June 2004 and 31 May 2005. Cases deemed to be ‘sensitive’ (e.g. a parental death) were removed from the sample 16.

### Variables

Supporting information provides detail on all variables (Table S1). Exposure to SSBs was measured at age 4–5 with the question: ‘How often does ^childname drink soft drinks, not including diet or sugar‐free drinks? INTERVIEWER: Please include diluting juice but do not include fresh fruit juice or water’, p. 26. Exposure to ASBs was measured at age 4–5 with the question: ‘How often does ^childname drink diet or low calorie soft drinks? INTERVIEWER: Include cans, bottles, mixers. Include diet or low‐cal flavoured water here. Do not include fresh fruit juice or water’, p. 26. Reported frequencies were recoded for analysis (Table S2).

Body mass index (BMI) was generated from heights and weights when the children were aged 7–8 16. Following a protocol, children were asked to remove shoes and socks, measurements were taken on a non‐carpeted surface, and only reliable measurements used 16. The British 1990 growth reference curves (the National BMI percentiles classification) were employed by the GUS project team to define cut‐offs for overweight and obesity (85th and 95th percentile, respectively) 16, 17. Original categories were recoded for analysis (Table S3).

Several covariates were selected when the children were aged 4–5 years. Self‐reported income was adjusted using an equivalence scale 16 (Table S4). Maternal educational level was based on self‐report according to the Scottish Credit and Qualifications Framework Category and recoded (Table S5), and Scottish Index of Multiple Deprivation was included. Consumption of breakfast, milk and water, composite measures of fruit and vegetables, sweets and crisps, processed meals, weekday television viewing time and a composite measure of physical activity was also included (Table S6). BMI at 3–4 years provided baseline BMI, and mothers' heights and weights were measured to derive maternal BMI (at 5–6 years).

### Statistical analysis

Cases were excluded where the respondent was not the child's mother (*n* = 61); there were missing data on exposure (*n* = 1) or outcome (*n* = 71); the respondent elected to skip physical activity questions because of the child's longstanding illness or disability (*n* = 2); outliers on BMI (*n* = 1) and television viewing (*n* = 5); and for cases with no longitudinal weight (who had not responded at every sweep) (*n* = 69) 16. The final sample (*n* = 2986) is shown in Fig. S1.

Equivalized income had 176 missing cases; however, manual imputation from Sweep 4 (age 3–4 years) and Sweep 1 (10 months) reduced this to 20 missing cases (0.7%). Baseline BMI had 205 missing cases (6.87%) and maternal BMI 401 missing cases (13.4%). Multivariate models were fitted for ‘complete cases’ (*n* = 2332).

Data analysis was executed using STATA Version 12 (Statistical Software: Release 12. College Station, TX: StataCorp LP 2011). The GUS project team modelled longitudinal weights using logistic regression 16, which were applied as probability weights. The analysis also accounted for clustering and stratification of the data. Pearson chi‐square tests were used for associations between exposure variables and covariates. Linear tests for trend were not possible on survey data, and so univariate logistic regressions were employed for ordered categorical variables. Multivariate logistic regression was employed, and confounding variables identified as relevant from existing literature were included in the analysis. As supplementary analyses, linear regressions were performed using BMI as a continuous outcome variable, and secondly, children categorized as obese at baseline were removed (*n* = 270), and linear regressions rerun.

### Ethics

Ethical approval was obtained for the first sweep of data collection from the Scotland ‘A’ MREC committee (Reference: 04/M RE 1 0/59), and subsequent sweeps were approved by the same committee by substantial amendment. This study was approved by the University of Glasgow MVLS Research Ethics Committee in February 2016 (Project No: 200150085).

## Results

**Figure 1:**
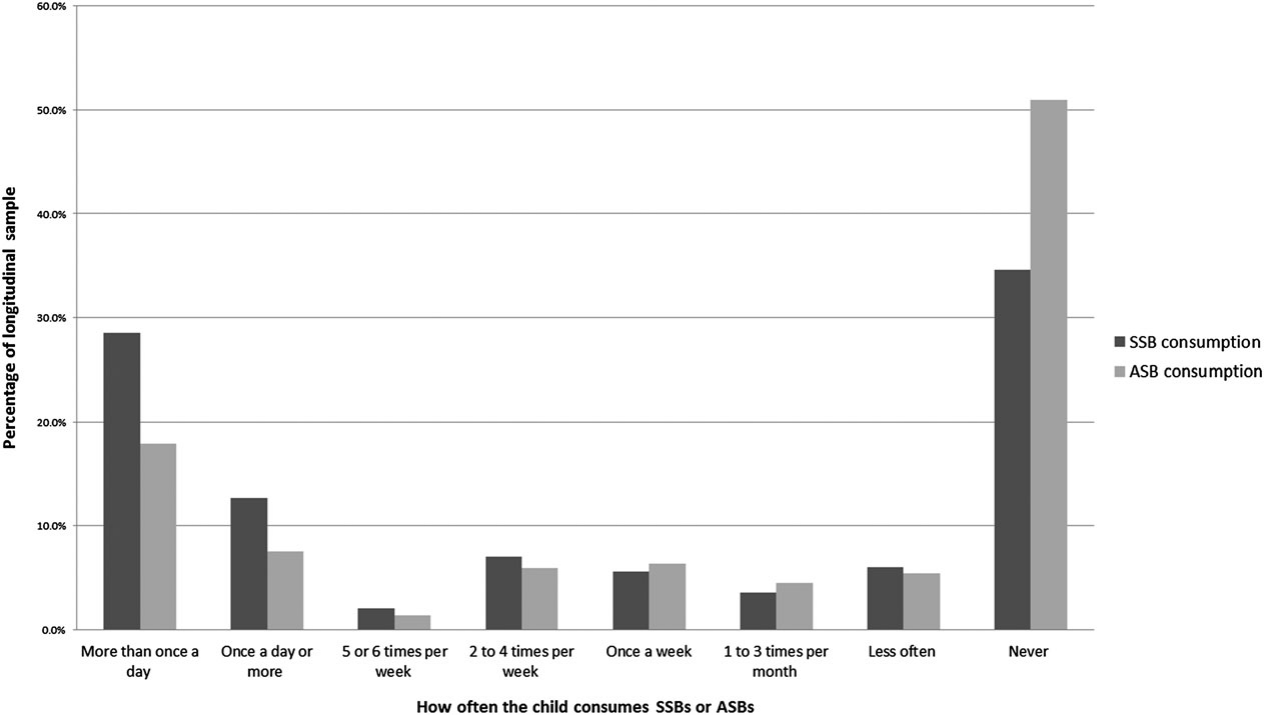
Histogram of sugar‐sweetened beverage consumption and artificially sweetened beverage consumption in longitudinal sample (*n* = 2986). ASB, artificially sweetened beverages; SSB, sugar‐sweetened beverages.

For both types of beverage, children at 4–5 years either consumed these drinks frequently (i.e. daily or more) or never (Fig. 1). More than a quarter of children (25.4%) in the longitudinal sample consumed ASBs daily or more, and 41.2% consumed SSBs daily or more (Table S7).

Table S7 provides the descriptive characteristics of the longitudinal sample. All measures of socioeconomic circumstances showed statistically significant associations such that lower equivalized income, lower maternal education and higher deprivation were each associated with higher prevalence of frequent consumption of both ASBs and SSBs. Several dietary variables were associated with beverage consumption. These included usually eating breakfast, eating more than five different types of fruit and vegetables in the previous day, drinking water once a day or more and eating sweets or crisps less than every day of the week, which were each negatively associated with both types of sweet beverage consumption. Watching less than 3 hours of television on weekdays was also negatively associated with ASB and SSB consumption. Baseline BMI and maternal BMI were each positively associated with ASB consumption but not SSB consumption.

The first analysis considered risk of overweight (including overweight and obese) at 7–8 years. There were no significant unadjusted associations between ASB consumption and overweight or SSB consumption and overweight, and this did not change for the adjusted models (Table 1) [Adjusted: ASB consumption 1–6 times per week odds ratio {OR} 1.02, 95% confidence interval {CI} 0.71–1.46; daily or more OR 0.85, 95% CI 0.63–1.15 and SSB consumption 1–6 times per week OR 0.94, 95% CI 0.68–1.30; daily or more OR 1.18, 95% CI 0.92–1.52]. Covariates significant in the models were baseline BMI, maternal BMI and the middle consumption category for processed meals.

**Table 1:** Unadjusted and adjusted multivariate logistic regression models for association between sugars‐sweetened/artificially sweetened beverage consumption at 4–5 years and overweight at 7–8 years (Reference category: ‘normal weight’ vs. ‘overweight including obese’). (Adjusted model complete cases *n* = 2332)

	Odds ratio	*P*‐value	(95% confidence interval)	Odds ratio	*P*‐value	(95% confidence interval)
*Unadjusted model*						
Beverage consumption	SSB consumption			ASB consumption		
<Once per week/never	Ref			Ref		
1 to 6 times per week	0.88	0.39	(0.66 – 1.18)	1.23	0.12	(0.95 – 1.60)
At least once a day	1.13	0.22	(0.93 – 1.38)	1.08	0.47	(0.88 – 1.32)
*Multivariate adjusted model*						
Beverage consumption	SSB consumption			ASB consumption		
<Once per week/never	Ref			Ref		
1 to 6 times per week	0.94	0.69	(0.68 – 1.30)	1.02	0.93	(0.71 – 1.46)
At least once a day	1.18	0.19	(0.92 – 1.52)	0.85	0.28	(0.63 – 1.15)
Child gender						
Male	Ref			Ref		
Female	1.33	0.02	(1.04 – 1.70)	1.34	0.02	(1.05 – 1.71)
Maternal age (years)						
20–29	Ref			Ref		
30–39	1.22	0.26	(0.86 – 1.74)	1.22	0.26	(0.86 – 1.75)
40+	1.13	0.58	(0.73 – 1.77)	1.11	0.63	(0.71 – 1.74)
Equivalized income						
Bottom quintile	Ref			Ref		
2nd quintile	1.10	0.63	(0.73 – 1.67)	1.11	0.61	(0.73 – 1.68)
3rd quintile	1.17	0.47	(0.76 – 1.80)	1.18	0.46	(0.76 – 1.81)
4th quintile	1.14	0.53	(0.75 – 1.71)	1.13	0.56	(0.75 – 1.70)
Top quintile	1.32	0.17	(0.89 – 1.96)	1.30	0.19	(0.87 – 1.95)
Maternal educational level						
No qualifications	Ref			Ref		
Other	1.55	0.43	(0.52 – 4.68)	1.48	0.49	(0.48 – 4.52)
Standard Grades/Intermediate vocational/Vocational	1.25	0.47	(0.67 – 2.33)	1.27	0.44	(0.69 – 2.35)
Higher grades and upper level vocational qualifications	0.96	0.89	(0.53 – 1.74)	0.95	0.87	(0.53 – 1.72)
Degree level academic/vocational qualifications	0.84	0.56	(0.45 – 1.54)	0.83	0.53	(0.45 – 1.51)
SIMD 2009 quintiles						
Least deprived quintile	Ref			Ref		
2nd quintile	1.01	0.93	(0.73 – 1.40)	1.02	0.90	(0.74 – 1.41)
3rd quintile	0.90	0.48	(0.66 – 1.22)	0.91	0.55	(0.66 – 1.24)
4th quintile	1.19	0.34	(0.83 – 1.72)	1.21	0.30	(0.84 – 1.73)
Most deprived quintile	1.16	0.47	(0.77 – 1.76)	1.18	0.43	(0.78 – 1.79)
Breakfast consumption						
Child usually eats breakfast	Ref			Ref		
Child does not usually eat breakfast	1.25	0.48	(0.68 – 2.29)	1.27	0.43	(0.69 – 2.36)
Fruit and vegetable consumption						
Ate less than 5 different types of fruit or vegetables yesterday	Ref			Ref		
Ate more than 5 different types of fruit or vegetables yesterday	0.89	0.39	(0.67 – 1.17)	0.88	0.35	(0.67 – 1.16)
Consumption of milk						
Drinks milk once a day or more	Ref			Ref		
Drinks milk less than every day of the week	1.14	0.33	(0.87 – 1.51)	1.15	0.31	(0.87 – 1.51)
Consumption of water						
Drinks water once a day or more	Ref			Ref		
Drinks water less than every day of the week	0.99	0.96	(0.76 – 1.29)	1.01	0.91	(0.78 – 1.32)
Consumption of sweets/crisps						
Eats sweets OR crisps less than every day of the week	Ref			Ref		
Eats sweets OR crisps once a day or more	1.15	0.29	(0.88 – 1.50)	1.18	0.23	(0.90 – 1.55)
Eats sweets AND crisps once a day or more	1.07	0.74	(0.69 – 1.67)	1.11	0.65	(0.71 – 1.72)
Consumption of processed meals						
Has not had processed meal in last 7 d	Ref			Ref		
Has had processed meal once in last 7 d	1.34	0.02	(1.05 – 1.70)	1.35	0.02	(1.05 – 1.72)
Has had processed meal twice or more past 7 d	0.96	0.80	(0.69 – 1.33)	0.97	0.84	(0.70 – 1.34)
Television viewing on weekdays						
Watches less than 3 h of TV on weekdays	Ref			Ref		
Watches more than 3 h of TV on weekdays	1.33	0.11	(0.93 – 1.91)	1.35	0.10	(0.95 – 1.94)
Physical activity time per week						
Does not meet physical activity guidelines (i.e. 420 min per week)	Ref			Ref		
Does meet physical activity guidelines (i.e. 420 min per week)	1.05	0.74	(0.79 – 1.39)	1.05	0.74	(0.79 – 1.39)
BMI at sweep 4 (age 3–4)						
Healthy weight (below 85th percentile)	Ref			Ref		
Overweight (at or above 85th percentile and below 95th percentile)	6.99	0.00	(5.07 – 9.65)	7.03	0.00	(5.10 – 9.69)
Obese (at or above 95th percentile)	26.57	0.00	(18.19 – 38.83)	26.44	0.00	(18.04 – 38.76)
Mother's BMI at sweep 6 (age 5–6)						
Underweight (<18.5)	Ref			Ref		
Healthy weight (18.5 to <25)	10.02	0.02	(1.58 – 63.39)	9.75	0.02	(1.48 – 64.43)
Overweight (25 to <30)	11.19	0.01	(1.70 – 73.74)	11.00	0.02	(1.59 – 75.94)
Obese (30 to <40)	20.35	0.00	(3.35 – 123.53)	19.98	0.00	(3.14 – 127.13)
Morbidly obese (40+)	29.17	0.00	(3.90 – 217.95)	28.86	0.00	(3.64 – 228.96)

ASB, artificially sweetened beverages; BMI, body mass index; SIMD, Scottish Index of Multiple Deprivation; SSB, sugar‐sweetened beverages.

The second analysis considered risk of obesity at 7–8 years. There were significant unadjusted associations between ASB consumption and risk of obesity (Table 2) (1 to 6 times per week OR 1.58, 95% CI 1.09–2.29; daily or more OR 1.32, 95% CI 1.06–1.64). CIs were wide, however, possibly due to the lower numbers in this group. Furthermore, prior to adjustment, children with the highest consumption had lower risk compared with weekly consumption (Table 2). In the adjusted model, the associations were attenuated such that children who consumed ASBs 1 to 6 times per week at 4–5 years had 1.57 times greater risk of obesity at 7–8 years (OR 1.57, 95% CI 1.05–2.36). The adjusted model continued to show a non‐linear pattern (daily or more OR 1.04, 95% CI 0.74–1.45), and only the middle category (1 to 6 times per week) remained significant. For SSB consumption, prior to adjustment, there was no significant association with obesity (1 to 6 times per week OR 1.04, 95% CI 0.74–1.45; daily or more OR 1.11, 95% CI 0.88–1.39). However, in the adjusted model, those who consumed SSBs 1 to 6 times per week had 1.65 times (95% CI 1.12–2.44) greater risk of obesity at 7–8 years, and the highest consumption category was not significant (OR 1.19, 95% CI 0.85–1.65). Again, wide CIs, lower numbers in the middle category and a non‐linear pattern suggest cautious interpretation. Baseline BMI, maternal BMI and breakfast consumption were significant covariates in both models (Table 2).

**Table 2:** Unadjusted and adjusted multivariate logistic regression models for association between sugar‐sweetened/artificially sweetened beverage consumption at 4–5 years and obesity at 7–8 years. (Reference category: ‘non obese’ vs. ‘obese’) (Adjusted model complete cases *n* = 2332)

	Odds ratio	*P*‐value	(95% confidence interval)	Odds ratio	*P*‐value	(95% confidence interval)
*Unadjusted model*						
Beverage consumption	SSB consumption			ASB consumption		
<Once per week/never	Ref			Ref		
1 to 6 times per week	1.04	0.83	(0.74 – 1.45)	1.58	0.02	(1.09 – 2.29)
At least once a day	1.11	0.36	(0.88 – 1.39)	1.32	0.01	(1.06 – 1.64)
*Multivariate adjusted model*						
Beverage consumption	SSB consumption			ASB consumption		
<Once per week/never	Ref			Ref		
1 to 6 times per week	1.65	0.01	(1.12 – 2.44)	1.57	0.03	(1.05 – 2.36)
At least once a day	1.19	0.30	(0.85 – 1.65)	1.04	0.84	(0.74 – 1.45)
Child gender						
Male	Ref			Ref		
Female	1.17	0.34	(0.85 – 1.63)	1.16	0.39	(0.83 – 1.61)
Maternal age (years)						
20–29	Ref			Ref		
30–39	1.50	0.13	(0.87 – 2.72)	1.45	0.08	(0.96 – 2.18)
40+	1.54	0.50	(0.79 – 1.61)	1.53	0.15	(0.86 – 2.71)
Equivalized income						
Bottom quintile	Ref			Ref		
2nd quintile	1.13	0.50	(0.79 – 1.61)	1.12	0.53	(0.78 – 1.61)
3rd quintile	0.97	0.90	(0.57 – 1.64)	0.96	0.87	(0.56 – 1.64)
4th quintile	1.09	0.74	(0.66 – 1.80)	1.07	0.79	(0.65 – 1.76)
Top quintile	1.01	0.97	(0.53 – 1.95)	1.01	0.98	(0.51 – 1.97)
Maternal educational level						
No qualifications	Ref			Ref		
Other	3.75	0.01	(1.31 – 10.73)	3.85	0.01	(1.34 – 11.11)
Standard Grades/Intermediate vocational/Vocational	1.35	0.44	(0.62 – 2.93)	1.33	0.44	(0.63 – 2.82)
Higher grades and upper level vocational qualifications	1.10	0.79	(0.55 – 2.18)	1.12	0.74	(0.58 – 2.17)
Degree level academic/vocational qualifications	0.97	0.94	(0.45 – 2.10)	0.98	0.95	(0.46 – 2.08)
SIMD 2009 quintiles						
Least deprived quintile	Ref			Ref		
2nd quintile	0.95	0.83	(0.62 – 1.47)	0.98	0.91	(0.64 – 1.49)
3rd quintile	1.15	0.48	(0.77 – 1.72)	1.11	0.61	(0.74 – 1.67)
4th quintile	1.19	0.49	(0.72 – 1.94)	1.16	0.54	(0.71 – 1.90)
Most deprived quintile	1.24	0.46	(0.70 – 2.19)	1.21	0.52	(0.68 – 2.14)
Breakfast consumption						
Child usually eats breakfast	Ref			Ref		
Child does not usually eat breakfast	2.17	0.02	(1.12 – 4.20)	2.14	0.03	(1.10 – 4.16)
Fruit and vegetable consumption						
Ate less than 5 different types of fruit or vegetables yesterday	Ref			Ref		
Ate more than 5 different types of fruit or vegetables yesterday	0.77	0.11	(0.55 – 1.06)	0.77	0.11	(0.55 – 1.07)
Consumption of milk						
Drinks milk once a day or more	Ref			Ref		
Drinks milk less than every day of the week	1.39	0.04	(1.02 – 1.89)	1.36	0.05	(1.01 – 1.84)
Consumption of water						
Drinks water once a day or more	Ref			Ref		
Drinks water less than every day of the week	1.07	0.70	(0.77 – 1.47)	1.07	0.69	(0.77 – 1.48)
Consumption of sweets/crisps						
Eats sweets OR crisps less than every day of the week	Ref			Ref		
Eats sweets OR crisps once a day or more	1.13	0.46	(0.82 – 1.55)	1.11	0.54	(0.80 – 1.54)
Eats sweets AND crisps once a day or more	0.96	0.86	(0.60 – 1.52)	0.96	0.86	(0.61 – 1.52)
Consumption of processed meals						
Has not had processed meal in last 7 d	Ref			Ref		
Has had processed meal once in last 7 d	1.22	0.18	(0.91 – 1.63)	1.19	0.24	(0.89 – 1.59)
Has had processed meal twice or more past 7 d	1.05	0.80	(0.71 – 1.55)	1.04	0.82	(0.71 – 1.54)
Television viewing on weekdays						
Watches less than 3 h of TV on weekdays	Ref			Ref		
Watches more than 3 h of TV on weekdays	1.40	0.16	(0.88 – 2.22)	1.41	0.13	(0.90 – 2.22)
Physical activity time per week						
Does not meet physical activity guidelines (i.e. 420 min per week	Ref			Ref		
Does meet physical activity guidelines (i.e. 420 min per week)	1.14	0.42	(0.83 – 1.57)	1.12	0.48	(0.81 – 1.55)
BMI at sweep 4 (age 3–4)						
Healthy weight (below 85th percentile)	Ref			Ref		
Overweight (at or above 85th percentile and below 95th percentile)	5.80	0.00	(3.83 – 8.80)	5.82	0.00	(3.87 – 8.75)
Obese (at or above 95th percentile)	26.90	0.00	(18.40 – 39.33)	26.15	0.00	(18.07 – 37.85)
Mother's BMI at sweep 6 (age 5–6)						
Underweight (<18.5)	Ref			Ref		
Healthy weight (18.5 to <25)	2.80	0.34	(0.34 – 23.28)	2.85	0.32	(0.36 – 22.54)
Overweight (15 to <30)	3.45	0.25	(0.41 – 29.30)	3.51	0.23	(0.44 – 28.03)
Obese (30 to <40)	6.62	0.07	(0.83 – 52.63)	6.63	0.07	(0.88 – 49.86)
Morbidly obese (40+)	10.50	0.05	(1.05 – 105.32)	10.44	0.04	(1.10 – 98.96)

ASB, artificially sweetened beverages; BMI, body mass index; SIMD, Scottish Index of Multiple Deprivation; SSB, sugar‐sweetened beverages.

Linear regressions showed that for SSB consumption, there was a significant association with BMI for the highest consumption category (daily or more 0.19, 95% CI 0.01–0.37), whilst for ASB consumption, there were no significant associations with BMI, although the middle category was approaching significance (1–6 times per week 0.30, 95% CI −0.01–0.61) (Table S8). When children who were categorized as obese at baseline were removed from the analysis (*n* = 270), there remained a significant association for the highest consumption category of SSB consumption (daily or more 0.27, 95% CI 0.12–0.43), and the associations for ASB consumption remained non‐significant (Table S9).

## Discussion

There was no evidence of significant associations between exposure to ASBs/SSBs at 4–5 years and risk of overweight at 7–8 years. The unadjusted association between ASB consumption at 4–5 years was associated with risk of obesity at 7–8 years; however, this did not follow a linear pattern, and only the middle category remained significant in the adjusted model. There were no significant unadjusted associations between SSB consumption at 4–5 years and risk of obesity at 7–8 years; although, adjustment showed a significant association for the middle consumption category. Linear regressions indicated a positive association between SSB consumption and BMI for the highest consumption category, but no significant relationship for ASB consumption, and this pattern did not change when children who were obese at baseline were removed from the analysis. Given these mixed findings, it is important to interpret them with caution. These results may indicate a truly mixed picture in terms of risk of obesity associated with ASB/SSB consumption, or they may reflect methodological challenges in measuring such associations (discussed below).

In this study, the prevalence of 4‐ to 5‐year‐olds consuming ASBs daily was 25% compared with 39% of 11‐year‐olds in a similar nationally representative longitudinal cohort study of children born in the UK, the Millennium Cohort Study 18. The prevalence of 4‐ to 5‐year‐olds consuming SSBs daily was 41% in this longitudinal Scottish sample compared with 31% of 11‐year‐olds in the Millennium Cohort Study 18. This demonstrates striking levels of daily consumption of ASBs and SSBs in under‐5s in Scotland.

The results are in line with research that has shown inconclusive evidence for SSB consumption and weight outcomes 19, and contrast with substantial evidence of SSBs as a risk factor for obesity 5. In relation to ASBs, the results build on pre‐existing mixed evidence by displaying similar results in a longitudinal sample 13, 20 but also add to recent evidence of a positive association between ASB consumption and change in body fat 18.

To the best of our knowledge, this is one of the first studies to prospectively examine the longer term impact of exposure to ASBs in early childhood. The longitudinal design, relatively large sample, and nationally representative cohort provides a robust source of data. GUS offers a rich dataset that permits inclusion of covariates relevant to wider aspects of children's lives. A further strength is precise outcome measurement, and weighting, clustering and stratification of the sample.

Nevertheless, GUS was not designed with a nutritional focus and did not quantify portion size or caloric intake 21, 22. Dietary information may be subject to self‐report bias, particularly under‐reporting 22, which may underestimate associations. Exposure misclassification is possible given no established definitions of sweetened beverages 6, 21. Residual confounding may also remain unaccounted for, such as overall diet quality or total energy intake 5. Bias may be introduced through dropout and complete case analysis due to missing data (e.g. baseline BMI and maternal BMI) that may misjudge associations 21, although weighting will likely account for this to some extent. Finally, the binary outcome (risk of childhood obesity/no risk) limits numbers for analysis; however, this outcome is relevant for public health.

The findings must be understood in the context of a high number of determinants of obesity 23. Each individual exposure may show a relatively weak association 21, 23, and exposure before age 5 may take years to demonstrate cumulative impact, which could explain why some associations are not significant 21. Indeed, it has been shown that high cumulative consumption of SSBs at the preschool stage increases the risk of obesity in later childhood and early adolescence 24. Furthermore, recent findings show that the proportion of children categorized as obese is relatively constant up to age 7, and a significant increase in obesity prevalence occurs between the ages of 7 and 11 25. Therefore, some (albeit mixed) evidence of associations between SSB/ASB consumption and risk of obesity at age 7–8 years may suggest the possibility that some children may be on a trajectory towards patterns of beverage consumption and weight gain, which may become increasingly evident later in childhood and adolescence 26.

The evidence presented here must be interpreted in relation to association, rather than proving causality 11, 23. Conclusively demonstrating the causal influence of ASBs is complicated due to reverse causality 27. Our analyses demonstrated some significant associations that suggest that ASBs and SSBs may be associated with obesity, even when accounting for baseline weight. However, it is possible that children who were obese at 4–5 years may have been encouraged to drink ASBs to reduce excess weight. When baseline BMI was not adjusted for, no effects were evident; thus, we cannot conclusively rule out reverse causality.

Contemporary obesity policies have focused on sugar, but there has been less consideration of ASBs 10, or overall sweetness (added sugars and artificial sweeteners) in children's diets 28. Recent research has shown that a sizeable proportion of drinks marketed to children contains artificial sweeteners 29 and that ASB consumption may be associated with higher sugar intake from solids for boys 30. Thus, there are concerns that replacing SSBs with ASBs may not fully address sugar intake 29, and it has been argued that without sufficient evidence, ASBs should not be promoted as a healthier alternative 10. Global trends indicate declining sales of SSBs in Western Europe, North America and Australasia, but twice the intake of ASBs in these countries compared with other regions 2. Evidence from the USA indicates increased purchases of food and beverages containing artificial sweeteners, or those containing *both* artificial and added sugars 9. Thus, the relative importance of ASBs in the global food system may be increasing, indicating the imperative for increased research and policy attention on these products 10.

Our study demonstrates the urgent need for long‐term longitudinal studies on ASB intake in childhood and weight outcomes later in life 13. Future longitudinal research should accurately measure both sugar‐sweetened and artificially sweetened products and address misclassification 21. Attending to both forms of sweeteners may be particularly important given recent trends towards product reformulation to include artificial sweeteners 9.

## Conclusions

This study demonstrated high prevalence of frequent consumption of both ASBs and SSBs at age 4–5 years. Longitudinal analysis to 7–8 years showed some evidence of associations between ASB/SSB consumption and obesity, but not overweight. Non‐linear effect sizes and significant results in the category with lowest numbers suggest that these results must be interpreted cautiously. Future research must continue to examine long‐term impacts of early exposure to sweetness (both sugary and artificial) in the liquids children consume 30.

## Conflict of interest statement

No conflict of interest was declared.

## Supporting information

Table S1: Variables selected for analysis
Table S2: Recoding of frequency of SSB/ASB consumption
Table S3: Recoding of BMI classifications to create binary variables for analysis
Table S4: Equivalence scales for different members of the household
Table S5: Recoding of maternal educational level variable
Table S6: Recoding of physical activity categories
Fig. S1: Flow diagram of the final longitudinal sample
Table S7: Descriptive characteristics of final longitudinal sample (sweeps 1, 4, 5, 6 and 7)
Table S8: Multivariate linear regression models for association between SSB/ASB consumption at 4–5 years and BMI at 7–8 years
Table S9: Multivariate linear regression models for association between SSB/ASB consumption at 4–5 years and BMI at 7–8 years with children categorized as obese at baseline removed
