# Supplementary material for: Exposure to liquid sweetness in early childhood: artificially‐sweetened and sugar‐sweetened beverage consumption at 4–5 years and risk of overweight and obesity at 7–8 years
Source: Pediatr Obes. 2018 Apr 6;13(12):755–65. doi: 10.1111/ijpo.12284 (PMC6492200; doi:10.1111/ijpo.12284)
Supplement: Supplementary file 1 — Table S1: Variables selected for analysis Table S2: Recoding of frequency of SSB/ASB consumption Table S3: Recoding of BMI classifications to create binary variables for analysis Table S4: Equivalence scales for different members of the household Table S5: Recoding of maternal educational level variable Table S6: Recoding of physical activity categories Fig. S1: Flow diagram of the final longitudinal sample Table S7: Descriptive characteristics of final longitudinal sample (sweeps 1, 4, 5, 6 and 7) Table S8: Multivariate linear regression models for association between SSB/ASB consumption at 4–5 years and BMI at 7–8 years Table S9: Multivariate linear regression models for association between SSB/ASB consumption at 4–5 years and BMI at 7–8 years with children categorized as obese at baseline removed [file IJPO-13-755-s001.pdf]

SUPPORTING INFORMATION

Exposure to liquid sweetness in early childhood: Artificially-sweetened and sugar-sweetened beverage consumption at 4-5 years and risk of overweight and obesity at 7-8 years

Anna K. Macintyre, Centre for Health Policy, University of Strathclyde

Louise Marryat, Farr Institute Scotland, University of Edinburgh

Stephanie Chambers, MRC/CSO Social & Public Health Sciences Unit, University of Glasgow

Address for correspondence: Dr Anna K. Macintyre, Centre for Health Policy, University of Strathclyde. Email: anna.macintyre@strath.ac.uk

Supporting Information

Table S1: Variables selected for analysis

Table S2: Recoding of frequency of SSB/ASB consumption

Table S3: Recoding of BMI classifications to create binary variables for analysis

Table S4: Equivalence scales for different members of the household

Table S5: Recoding of maternal educational level variable

Table S6: Recoding of physical activity categories

Figure S1: Flow diagram of the final longitudinal sample

Table S7: Descriptive characteristics of final longitudinal sample (sweeps 1, 4, 5, 6 and 7)

Table S8: Multivariate linear regression models for association between SSB/ASB consumption at 4-5 years and BMI at 7-8 years

Table S9: Multivariate linear regression models for association between SSB/ASB consumption at 4-5 years and BMI at 7-8 years with children categorised as obese at baseline removed

Additional information on variables and re-coding

'Growing Up in Scotland' collects data on a range of areas including social, emotional and cognitive development, physical health and mental wellbeing, family and community circumstances, employment and childcare, and educational experiences [1]. At each sweep, a slightly different mixture of questions was asked. Full details on the data collected at each sweep are available from the GUS Topic Guide: http://growingupinscotland.org.uk/using-gus-data/data-documentation/, accessed on 23rd May 2016 [2]. Table S2 below outlines the variables selected for this study.

Table S1: Variables selected for analysis

Type of variable	Measurement
Exposure	Carer report of frequency of SSB consumption
	Carer report of frequency of ASB consumption
Outcome	BMI classification based on National BMI percentiles
Covariates	*Socio-demographic factors:* child gender; maternal age; household equivalised household income; maternal educational level; Scottish Index of Multiple Deprivation
	Dietary factors: breakfast consumption; water/milk consumption; fruit/vegetable consumption*; sweets/crisps consumption*; processed meals consumption*.
	Activity factors: TV viewing time (weekdays); measure of weekly physical activity level*.
	BMI factors: BMI measured at sweep 4; maternal BMI
	*composite variables composed of 2 or more variables

Data collection at each sweep was intended to be 6 weeks before the child's next birthday, and so the exposure was measured when the children were just under 5 years (i.e. 4-5 years), and the outcome when the children were just under 8 years (i.e. 7-8 years).

- Exposure Variables

For both exposures, respondents were asked to report frequency of consumption according to the following categories:

1. More than once a day
2. Once a day
3. 5 or 6 times a week
4. 2 to 4 times a week
5. Once a week
6. 1 to 3 times per month
7. Less often
8. Never

For analytic purposes it was necessary to re-categorise the exposure variables[3].

Table S2: Recoding of frequency of sugar-sweetened/artificially-sweetened beverage consumption

Original category from questionnaire	Re-coded into 3 categories
**1.** More than once a day	At least once a day
**2.** Once a day	
**3.** 5 or 6 times a week	1 to 6 times per week
**4.** 2 to 4 times a week	
**5.** Once a week	
**6.** 1 to 3 times per month	< once per week/never
**7.** Less often	
**8.** Never	

- Outcome variable

Firstly, BMI at age 3-4 years and age 7-8 years was recoded because for the purposes of these analyses underweight was considered similar to healthy weight i.e. not overweight. In order to be able to consider overweight and obesity separately, the BMI outcome variable at age 7-8years was recoded into two binary variables, as shown in Table 3 below.

Table S3: Recoding of body mass index classifications to create binary variables for analysis

Original categories from National BMI percentiles classification	Recoded BMI categories	Recoded into binary variable for analysis of overweight	Recoded into binary variable for analysis of obesity
Underweight	Healthy weight	Normal weight	Non-obese
Normal weight			
Overweight (85th percentile)	Overweight	Overweight (including obese)	
Obese (95th percentile)	Obese		Obese

A wide range of risk factors have been linked to obesity [4-6]. Although it was not possible to include all potential risk factors, a range of confounding variables were selected for inclusion based on some of the types variables which have been found to be significant in previous studies [3,7,8].

- Sociodemographic variables

Equivalised income. Respondents were asked to report the *"total income of your household from all sources before tax – including benefits, interest from savings and so on"* ([9]p.36). Respondents were provided with a list of 17 income bands, from *"Less than £3,999 pa"* to *"£56,000 or more pa"* ([9]p.36). These income bands were then adjusted by the GUS project team using an equivalence scale, as show in Table S4 below, to provide "equivalised annual household income", and this was also split into quintiles ([10]p.20). This adjustment takes into account that the standard of living that a family can achieve will differ depending on the size of the household and the ratio of adults and children [10].

Table S4: Equivalence scales for different members of the household (adapted from sweep 5 user guide) [10]

**Member of household**	**Equivalence scale**
Head	0.67
Subsequent adults	0.33
Each child aged 0 to 13 years	0.20
Each child aged 14 to 18 years	0.33

Maternal educational level. At sweep 1, respondents were provided with a list of educational examinations/qualifications, and asked to identify those that applied to them. At subsequent sweeps respondents were also asked if they had achieved any new qualifications. This information was used by the project team to develop a derived variable *"Highest educational level of respondent"*[9]p.131. For the purposes of this study, educational level was based on a derived variable at sweep 5. Only cases where the respondent was the child's mother (including step mothers and adoptive mothers) were included. For the purposes of our study we wanted to be able to control for maternal educational level (and maternal BMI) which have been shown to be relevant determinants of child BMI[8]. Therefore we wished to include these variables as covariates. If we had included respondents who were not the child's mother this would have prevented being able to control specifically for maternal factors – the variable would instead have been 'respondent/carer educational level' (and 'respondent/carer BMI'), which would not have provided the required specificity. This variable is therefore understood as *maternal* educational level. The derived variable coded the qualifications that the respondent had achieved according to the Scottish Credit and Qualifications Framework as outlined in Table S5 below. These were recoded in order to simplify categories for analytic purposes.

Table S5: Recoding of maternal educational level variable

**Scottish Credit and Qualifications Framework category**	**Recoded categories**
Information not available	Information not available
No qualification	No qualification
Other	Other
Lower level standard grades and vocational qualifications	Standard grade and vocational or intermediate vocational qualifications
Upper level standard grades and intermediate vocational qualifications	

Higher grades and upper level vocational qualifications	Higher grades and upper level vocational qualifications
Degree level academic and vocational qualifications	Degree level academic and vocational qualifications

- Dietary variables

Breakfast consumption: Respondents were asked: *"On a typical day, does ^childname eat breakfast?" Respondents could answer Yes or No*. [9]p.23 and this was coded as child usually eats breakfast or does not usually eat breakfast.

Fruit and vegetable consumption: Respondents were asked: *"How many different types of vegetable did ^childname eat yesterday? RANGE 0-10 and "How many different types of fruit did ^childname eat yesterday? RANGE 0 -10* [9]p.24.

These data were combined to create a variable for the total number of different types of fruit and vegetables consumed in the previous day. The Scottish Government's Dietary Goals recommend that adults and children eat 5 portions of fruit and vegetables every day [11]. This variable was categorised into two groups i.e. those children who ate 5 or more different types of fruit and vegetables in the previous day, and those who ate less than 5 types of fruit and vegetables per day. It is recognised that the question asks about the number of different *types* of fruits and vegetables, not number of portions, and this is a limitation of the information derived from this variable.

Milk and water consumption: Respondents were asked to report on the child's consumption of other beverages: *"How often does ^childname drink milk, not including milkshakes or other flavoured milks? INTERVIEWER: include soya/goat's milk"* [9]p. 26, and *"How often does ^childname drink un-flavoured water, for example, from the tap, a water cooler or a bottle of water?"* [9] p.26. Respondents were asked to choose from the eight categories of frequency as outlined above and these were recoded into the binary category of either daily or less than daily consumption.

Sweets and crisps consumption: Respondents were asked: "*How often does ^childname eat sweets or chocolates? INTERVIEWER: Include only whole packets of sweets or a chocolates/chocolate bar, not individual sweets"* and *"How often does ^childname eat crisps?"* [9] p.25.

These variables were recoded into binary variables i.e. whether the child ate sweets/chocolates once a day or more, versus less than once a day. The same categorisation was used for the crisps variable. These variables were then combined into three categories:

0 = "Eats sweets OR crisps less than every day of the week"
1 = "Eats sweets OR crisps once a day or more"
2 = "Eats sweets AND crisps once a day or more"

Processed meals consumption: Respondents were also asked to report on the child's consumption of different types of meals. Three of these questions were chosen for their relevance to overall diet quality: "*Can you tell me on how many days in the last week ^childname has had each of the following things for ^his main meal? By 'the last week', I mean the last 7 days. (RANGE 0-7)*

… a ready meal?
… a take-away meal, for example, from a fish and chip shop or an Indian or Chinese takeaway?
… a fast-food meal, for example, from McDonald's?" [9] p.22.

The scores for each of these three variables were then summed to give the frequency of total consumption. A new variable was created according to the following categories:

0 = "Has not had processed meal in last 7 days"
1 = "Has had processed meal once in last 7 days"
2 = "Has had processed meal twice or more in last 7 days"

- Activity variables

Respondents were asked a range of questions regarding the child's physical activity, television and computer use.

Television viewing: Several questions asked respondents about the child's television viewing, including patterns for both weekday and weekends. The following question was chosen as a measure of TV viewing time:

"How long would *^childname usually watch television for in total on an average weekday? INTERVIEWER PLEASE ENTER TIME IN HOURS* (Range 0-24) [9]p. 84.

In line with previous analysis of GUS data, TV viewing was categorised into those that watched less than 3 hours of TV per day or more than 3 hours per day [12]. The weekday variable was chosen because the distributions for the weekday and weekend variables were similar, and therefore only one was required.

Physical activity: Respondents were asked a range of questions regarding the child's physical activity. Firstly respondents were asked to report on whether a child had engaged in a range of different activities in the previous week. Then respondents were asked to report the time spent on each activity:

"Now looking at this card, in the last week, how much time did ^childname spend doing activity?

1. *Less than 15 minutes*
2. *15 minutes, less than 30 minutes*
3. *30 minutes, less than 1 hour*
4. *1 hour, less than 2 hours*
5. *2 hours, less than 3 hours*
6. *3 hours or more*
7. *(No time – spontaneous code)"* [9]p.89.

This question was asked in relation to the time spent engaging in the following activities: riding a bicycle; throwing or kicking a ball; dancing or gymnastics; running and/or jumping; playing on a trampoline; swimming; playing at a soft play area or ball swamp; playing at a play/swing park; walking and doing something else active.

In order that a judgement could be made regarding a child's overall physical activity, these variables were combined. To generate a composite measure of physical activity, a decision was made to treat each category as the maximum number of minutes for that category, as shown in Table S6 below.

Table S6: Recoding of physical activity categories

**Original coding**	**New coding**
Less than 15 minutes	14 minutes
15 minutes, less than 30 minutes	29 minutes
30 minutes, less than 1 hour	59 minutes
1 hour, less than 2 hours	119 minutes
2 hours, less than 3 hours	179 minutes
3 hours or more	180 minutes
(No time – spontaneous code)	0 minutes

If it was recorded that this item was not applicable (i.e. the child had not taken part in that activity in the past week) or there was no time, then it was recorded as zero minutes. The minutes for each activity were then summed to generate a total number of minutes of physical activity for the previous week. The UK Government recommends that children and young people are active for 60 minutes per day, on every day of the week, and that children under 5 are active for 180 minutes per day [13]. The guideline for under 5s includes activities "of any intensity" [13]whilst the guideline for children and young people are focused on "moderate to vigorous intensity physical activity"[14]. The activities measured in GUS arguably fit with moderate to vigorous intensities, and so the guidelines for children and young people were used to inform interpretation of the data. It was not possible to calculate a meaningful measure of *daily* activity. Therefore the total number of minutes per week was compared to what would be achieved if a child was active for 60 minutes on every day of the week i.e. 420 minutes per week. This generated a binary variable:

0 = "Does not meet weekly activity guidelines (i.e. 420 minutes per week)"
1 = "Meets weekly activity guidelines of 60 mins per day (i.e. 420 minutes per week)"

It is acknowledged that this variable is a less than optimal measure of physical activity and it is likely that it does not represent actual activity levels. However it was reasoned that this was the best option for a proxy measure based on available data.

- BMI factors

Heights and weights were measured at sweep 4 (when the children were approximately 3 years), and the National (UK) BMI percentiles were calculated by the GUS project team[15]. National BMI percentile classification at age 3-4 years were used as a measure of baseline BMI.

Mothers' heights and weights were measured by trained interviewers at sweep 6 according to a protocol. Maternal BMI was then derived. It must be noted that estimated weights where the mother's weight was over 130kg were also included (At Sweep 6 this was 9 cases) (Growing Up in Scotland Main Carer Questionnaire Sweep 6).

Figure S1: Flow diagram of the final longitudinal sample

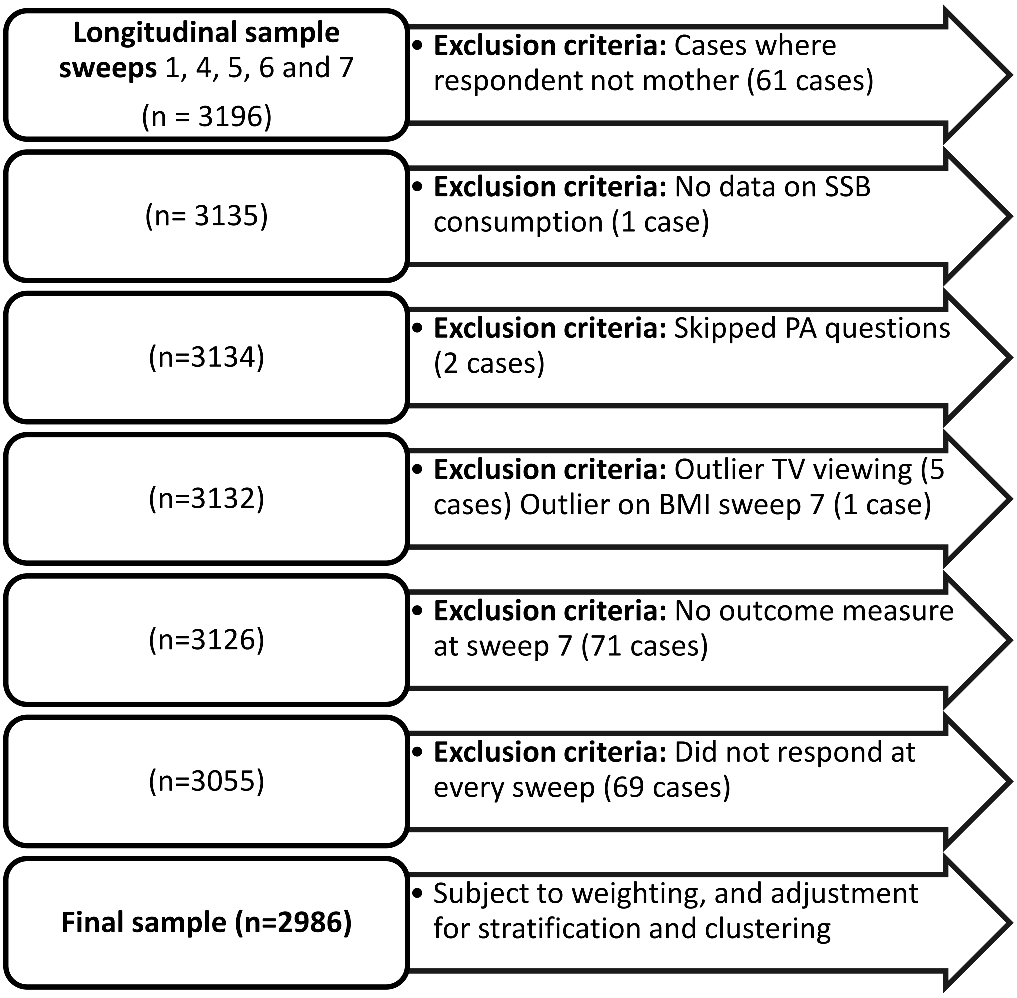

Table S7: Descriptive characteristics of final longitudinal sample (sweeps 1, 4, 5, 6 and 7) complete cases

		SSB consumption				ASB consumption		
**Variable**	**Total weighted proportions %**	**<Once per week/ never %**	**1 to 6 times per week %**	**At least once a day%**	**Variable**	**<Once per week / never%**	**1 to 6 times per week %**	**At least once a day %**
**SSB or ASB consumption**		44.2	14.7	41.2%	**SSB or ASB consumption**	61.0	13.6	25.4
**Child gender**					**Child gender**			
Male	51.1	50.5	56.4	49.7	Male	50.9	49.1	52.6
Female	48.9	49.5	43.6	50.3	Female	49.1	50.9	47.5
**Maternal age***					**Maternal age****			
20-29 years	24.1	22.0	22.6	26.9	20-29 years	21.9	28.3	27.0
30-39 years	55.6	56.1	51.8	56.4	30-39 years	56.2	52.2	55.8
40+ years	20.3	22.0	25.6	16.7	40+ years	21.8	19.5	17.2
**Equivalised income*****					**Equivalised income*****			
Bottom quintile	25.0	22.1	23.7	28.5	Bottom quintile	22.4	27.3	30.0
2nd quintile	24.1	22.9	20.0	26.8	2nd quintile	22.4	27.3	26.6
3rd quintile	17.0	16.9	18.6	16.6	3rd quintile	17.0	15.1	18.1
4th quintile	20.3	22.3	22.4	17.4	4th quintile	21.8	18.8	17.3
Top quintile	13.7	15.9	15.3	10.8	Top quintile	16.5	11.5	8.1
**Maternal educational level*****					**Maternal educational level*****			
No qualifications	8.1	6.0	8.3	10.3	No qualifications	6.2	14.9	9.2
Other	1.0	1.2	1.6	0.5	Other	0.8	1.7	0.9
Standard Grades/Intermediate vocational/Vocational	30.1	27.6	24.1	35.0	Standard Grades/Intermediate vocational/Vocational	26.6	29.5	38.8

Higher grades and upper level vocational qualifications	33.1	31.6	33.9	34.4	Higher grades and upper level vocational qualifications	34.0	30.6	32.3
Degree level academic/vocational qualifications	27.7	33.6	32.2	19.9	Degree level academic/vocational qualifications	32.4	23.3	18.8
**SIMD 2009 quintiles***					**SIMD 2009 quintiles*****			
Least deprived quintile	19.3	22.3	20.4	15.7	Least deprived quintile	23.1	13.8	13.0
2nd quintile	19.9	20.1	25.5	17.6	2nd quintile	21.1	16.0	19.0
3rd quintile	19.1	18.7	20.2	19.2	3rd quintile	18.5	19.2	20.6
4th quintile	18.9	18.4	16.4	20.2	4th quintile	16.8	21.9	22.2
Most deprived quintile	22.9	20.6	17.5	27.2	Most deprived quintile	20.6	29.1	25.2
**Breakfast consumption***					**Breakfast consumption****			
Child usually eats breakfast	95.1	96.5	94.7	93.8	Child usually eats breakfast	96.5	93.4	92.7
Child does not usually eat breakfast	4.9	3.5	5.3	6.2	Child does not usually eat breakfast	3.5	6.6	7.3
**Fruit and vegetable consumption******					**Fruit and vegetable consumption****			
Ate less than 5 different types of fruit or vegetables yesterday	69.0	65.0	65.7	74.5	Ate less than 5 different types of fruit or vegetables yesterday	66.5	73.8	72.5
Ate more than 5 different types of fruit or vegetables yesterday	31.0	35.0	34.3	25.5	Ate more than 5 different types of fruit or vegetables yesterday	33.5	26.3	27.5
**Consumption of milk*****					**Consumption of milk**			
Drinks milk once a day or more	69.5	71.3	74.6	65.7	Drinks milk once a day or more	69.7	68.6	69.6
Drinks milk less than every day of the week	30.5	28.7	25.4	34.3	Drinks milk less than every day of the week	30.3	31.4	30.4
**Consumption of water******					**Consumption of water*****			
Drinks water once a day or	65.3	70.6	70.2	57.9	Drinks water once a day or	69.1	62.0	58.1

more					more			
Drinks water less than every day of the week	34.7	29.4	29.8	42.1	Drinks water less than every day of the week	30.9	38.0	41.9
**Consumption of sweets/crisps*** **					**Consumption of sweets/crisps*** **			
Eats sweets OR crisps less than every day of the week	45.8	51.2	52.1	37.9	Eats sweets OR crisps less than every day of the week	50.1	41.3	38.0
Eats sweets OR crisps once a day or more	36.0	33.3	33.0	40.0	Eats sweets OR crisps once a day or more	34.4	38.0	38.6
Eats sweets AND crisps once a day or more	18.2	15.6	14.9	22.2	Eats sweets AND crisps once a day or more	15.5	20.7	23.4
**Consumption of processed meals*** **					**Consumption of processed meals***			
Has not had processed meal in last 7 days	40.7	45.7	45.3	33.5	Has not had processed meal in last 7 days	42.8	31.0	40.6
Has had processed meal once in last 7 days	35.6	34.6	31.8	38.2	Has had processed meal once in last 7 days	34.6	42.7	34.3
Has had processed meal twice or more past 7 days	23.7	19.7	22.9	28.3	Has had processed meal twice or more past 7 days	22.6	26.3	25.2
**Television viewing on weekdays** **					**Television viewing on weekdays***			
Watches less than 3 hours of TV on weekdays	85.9	87.8	89.2	82.5	Watches less than 3 hours of TV on weekdays	87.7	84.3	82.2
Watches more than 3 hours of TV on weekdays	14.2	12.2	10.8	17.5	Watches more than 3 hours of TV on weekdays	12.3	15.7	17.8
**Physical activity time per week**					**Physical activity time per week**			
Does not meet physical activity guidelines (i.e. 420 mins per week)	36.1	34.2	35.6	38.4	Does not meet physical activity guidelines (i.e. 420 mins per week)	36.5	35.6	35.3

Does meet physical activity guidelines (i.e. 420 mins per week)	63.9		65.8	64.5	61.6	Does meet physical activity guidelines (i.e. 420 mins per week)	63.5	64.4	64.7
**BMI at age 3-4 years**						**BMI at sweep 4***			
Healthy weight	73.6		73.2	74.9	73.6	Healthy weight	75.2	72.2	70.5
Overweight	15.7		15.4	15.4	16.1	Overweight	15.2	15.5	17.0
Obese	10.7		11.4	9.7	10.3	Obese	9.5	12.3	12.6
**Mother's BMI at child age 5-6 years**						**Mother's BMI at sweep 6*** **			
Underweight	1.1		1.2	0.0	1.4	Underweight	1.3	0.7	0.9
Healthy weight	42.0		41.9	45.4	40.8	Healthy weight	46.2	38.1	34.2
Overweight	30.9		29.8	33.0	31.4	Overweight	30.3	32.7	31.3
Obese	22.3		23.6	18.4	22.3	Obese	19.4	24.0	28.2
Morbidly obese	3.7		3.5	3.2	4.1	Morbidly obese	2.8	4.5	5.4
**BMI at age 7-8 years**						**BMI at sweep 7***			
Healthy weight	71.7		72.4	74.8	69.8	Healthy weight	72.7	68.4	71.2
Overweight	13.6		13.5	10.6	14.7	Overweight	14.3	12.5	12.4
Obese	14.8		14.1	14.6	15.5	Obese	13.0	19.2	16.5

*** <0.05; ** <0.01; ***<0.001; ****<0.0001**

Table S8: Multivariate adjusted linear regression models for association between sugar-sweetened/artificially-sweetened beverage consumption at 4-5 years and BMI at 7-8 years

Multivariate adjusted model	Coefficient	P-value	95% confidence interval		Coefficient	P-value	95% confidence interval	
**Beverage consumption**	**SSB consumption**				**ASB consumption**			
<once per week/never	Ref				Ref			
1 to 6 times per week	0.06	0.59	-0.17	0.29	0.30	0.06	-0.01	0.61
At least once a day	0.19	0.04	0.01	0.37	-0.11	0.34	-0.32	0.11
**Child gender**								
Male	Ref				Ref			
Female	0.49	0.00	0.30	0.67	0.49	0.00	0.30	0.68
**Maternal age**								
20-29 years	Ref				Ref			
30-39 years	0.12	0.44	-0.19	0.43	0.13	0.41	-0.18	0.43
40+ years	0.11	0.59	-0.30	0.52	0.11	0.59	-0.30	0.52
**Equivalised income**								
Bottom quintile	Ref				Ref			
2nd quintile	0.09	0.57	-0.23	0.42	0.09	0.58	-0.24	0.42
3rd quintile	0.19	0.34	-0.20	0.58	0.18	0.34	-0.20	0.57
4th quintile	0.25	0.14	-0.08	0.57	0.23	0.16	-0.09	0.55
Top quintile	0.23	0.15	-0.09	0.54	0.21	0.18	-0.10	0.53
**Maternal educational level**								
No qualifications	Ref				Ref			
Other	2.15	0.10	-0.41	4.71	2.15	0.10	-0.43	4.73
Standard Grades/Intermediate vocational/Vocational	-0.06	0.87	-0.85	0.72	-0.03	0.93	-0.79	0.72
Higher grades and upper level vocational qualifications	-0.33	0.39	-1.11	0.44	-0.31	0.42	-1.06	0.44
Degree level academic/vocational qualifications	-0.36	0.33	-1.11	0.38	-0.35	0.33	-1.08	0.37

**SIMD 2009 quintiles**								
Least deprived quintile	Ref				Ref			
2nd quintile	0.08	0.40	-0.11	0.27	0.08	0.37	-0.10	0.27
3rd quintile	0.08	0.50	-0.15	0.30	0.08	0.50	-0.15	0.30
4th quintile	0.11	0.43	-0.16	0.37	0.11	0.44	-0.17	0.38
Most deprived quintile	0.22	0.19	-0.12	0.57	0.22	0.21	-0.13	0.57
**Breakfast consumption**								
Child usually eats breakfast	Ref				Ref			
Child does not usually eat breakfast	0.60	0.08	-0.07	1.27	0.61	0.06	-0.04	1.26
**Fruit and vegetable consumption**								
Ate less than 5 different types of fruit or vegetables yesterday	Ref				Ref			
Ate more than 5 different types of fruit or vegetables yesterday	-0.09	0.30	-0.27	0.09	-0.10	0.29	-0.27	0.08
**Consumption of milk**								
Drinks milk once a day or more	Ref				Ref			
Drinks milk less than every day of the week	0.11	0.23	-0.07	0.30	0.11	0.23	-0.07	0.30
**Consumption of water**								
Drinks water once a day or more	Ref				Ref			
Drinks water less than every day of the week	-0.01	0.97	-0.26	0.25	0.01	0.94	-0.24	0.26
**Consumption of sweets/crisps**								
Eats sweets OR crisps less than every day of the week	Ref				Ref			
Eats sweets OR crisps once a day or more	0.08	0.43	-0.12	0.29	0.09	0.38	-0.12	0.30
Eats sweets AND crisps once a day or more	0.14	0.34	-0.15	0.44	0.16	0.27	-0.13	0.46
**Consumption of processed meals**								
Has not had processed meal in last 7 days	Ref				Ref			
Has had processed meal once in last 7 days	0.15	0.12	-0.04	0.33	0.14	0.15	-0.05	0.32
Has had processed meal twice or more past 7 days	0.09	0.50	-0.16	0.33	0.09	0.48	-0.16	0.34
**Television viewing on weekdays**								
Watches less than 3 hours of TV on weekdays	Ref				Ref			

Watches more than 3 hours of TV on weekdays	0.13	0.43	-0.20	0.46	0.15	0.36	-0.17	0.48
**Physical activity time per week**								
Does not meet physical activity guidelines (i.e. 420 mins per week)	Ref				Ref			
Does meet physical activity guidelines (i.e. 420 mins per week)	0.23	0.02	0.05	0.42	0.23	0.02	0.04	0.42
**BMI at age 3-4 years**								
Healthy weight	Ref				Ref			
Overweight	2.05	0.00	1.80	2.30	2.06	0.00	1.81	2.31
Obese	4.29	0.00	3.75	4.84	4.28	0.00	3.74	4.83
**Mother's BMI at child age 5-6 years**								
Underweight	Ref				Ref			
Healthy weight	0.77	0.01	0.23	1.31	0.76	0.01	0.22	1.29
Overweight	0.97	0.00	0.40	1.55	0.96	0.00	0.41	1.51
Obese	1.44	0.00	0.93	1.95	1.43	0.00	0.93	1.92
Morbidly obese	2.45	0.00	1.58	3.32	2.43	0.00	1.56	3.30

Table S9: Multivariate adjusted linear regression models for association between sugar-sweetened/artificially-sweetened beverage consumption at 4-5 years and BMI at 7-8 years with children categorised as obese at baseline removed

Multivariate adjusted model	Coefficient	P-value	95% confidence interval		Coefficient	P-value	95% confidence interval	
**Beverage consumption**	**SSB consumption**				**ASB consumption**			
<once per week/never	Ref				Ref			
1 to 6 times per week	0.00	0.98	-0.26	0.26	0.18	0.20	-0.10	0.45
At least once a day	0.27	0.00	0.12	0.43	-0.07	0.44	-0.25	0.11
**Child gender**								
Male	Ref				Ref			
Female	0.19	0.05	0.00	0.37	0.19	0.05	0.00	0.38
**Maternal age**								
20-29 years	Ref				Ref			
30-39 years	0.15	0.22	-0.09	0.40	0.15	0.22	-0.09	0.40
40+ years	0.13	0.41	-0.18	0.44	0.11	0.47	-0.20	0.42
**Equivalised income**								
Bottom quintile	Ref				Ref			
2nd quintile	0.09	0.57	-0.22	0.39	0.09	0.55	-0.22	0.41
3rd quintile	0.04	0.78	-0.25	0.33	0.05	0.75	-0.25	0.34
4th quintile	0.17	0.25	-0.12	0.46	0.16	0.28	-0.13	0.46
Top quintile	0.20	0.15	-0.07	0.47	0.20	0.17	-0.08	0.48
**Maternal educational level**								
No qualifications	Ref				Ref			
Other	0.37	0.51	-0.74	1.49	0.32	0.57	-0.79	1.42
Standard Grades/Intermediate vocational/Vocational	0.29	0.21	-0.17	0.75	0.30	0.19	-0.16	0.76
Higher grades and upper level vocational qualifications	0.06	0.76	-0.34	0.47	0.07	0.74	-0.34	0.48
Degree level academic/vocational qualifications	-0.04	0.82	-0.41	0.32	-0.06	0.76	-0.42	0.31

**SIMD 2009 quintiles**								
Least deprived quintile	Ref				Ref			
2nd quintile	0.14	0.18	-0.07	0.36	0.15	0.18	-0.07	0.36
3rd quintile	0.15	0.10	-0.03	0.34	0.15	0.10	-0.03	0.33
4th quintile	0.22	0.11	-0.05	0.50	0.23	0.10	-0.05	0.51
Most deprived quintile	0.42	0.01	0.09	0.74	0.43	0.01	0.10	0.76
**Breakfast consumption**								
Child usually eats breakfast	Ref				Ref			
Child does not usually eat breakfast	0.74	0.01	0.16	1.31	0.74	0.01	0.16	1.32
**Fruit and vegetable consumption**								
Ate less than 5 different types of fruit or vegetables yesterday	Ref				Ref			
Ate more than 5 different types of fruit or vegetables yesterday	0.00	0.96	-0.20	0.19	-0.01	0.90	-0.21	0.18
**Consumption of milk**								
Drinks milk once a day or more	Ref				Ref			
Drinks milk less than every day of the week	-0.06	0.49	-0.23	0.11	-0.05	0.57	-0.22	0.12
**Consumption of water**								
Drinks water once a day or more	Ref				Ref			
Drinks water less than every day of the week	-0.02	0.89	-0.24	0.20	0.01	0.96	-0.21	0.22
**Consumption of sweets/crisps**								
Eats sweets OR crisps less than every day of the week	Ref				Ref			
Eats sweets OR crisps once a day or more	0.10	0.37	-0.12	0.33	0.13	0.27	-0.10	0.35
Eats sweets AND crisps once a day or more	-0.03	0.85	-0.29	0.24	0.00	0.99	-0.26	0.26
**Consumption of processed meals**								
Has not had processed meal in last 7 days	Ref				Ref			
Has had processed meal once in last 7 days	0.22	0.02	0.03	0.40	0.23	0.02	0.04	0.41
Has had processed meal twice or more past 7 days	-0.04	0.69	-0.21	0.14	-0.02	0.81	-0.20	0.15
**Television viewing on weekdays**								
Watches less than 3 hours of TV on weekdays	Ref				Ref			

Watches more than 3 hours of TV on weekdays	0.16	0.41	-0.22	0.53	0.18	0.34	-0.20	0.56
**Physical activity time per week**								
Does not meet physical activity guidelines (i.e. 420 mins per week)	Ref				Ref			
Does meet physical activity guidelines (i.e. 420 mins per week)	0.27	0.01	0.08	0.47	0.27	0.01	0.08	0.47
**Mother's BMI at child age 5-6 years**								
Underweight	Ref				Ref			
Healthy weight	0.99	0.00	0.45	1.54	0.98	0.00	0.44	1.51
Overweight	1.20	0.00	0.70	1.70	1.18	0.00	0.69	1.67
Obese	1.64	0.00	1.11	2.18	1.62	0.00	1.09	2.15
Morbidly obese	2.12	0.00	1.19	3.05	2.11	0.00	1.19	3.03

References for Supporting Information

1. Bradshaw P, Corbett, J., Tiping, S. Growing Up in Scotland Sweep 6: 2010-2011 User Guide. Scottish Centre for Social Research nd.
2. Scottish Centre for Social Research. Growing Up in Scotland Topic Guide http://growingupinscotland.org.uk/using-gus-data/data-documentation/. Accessed 23rd May 2016
3. Laverty AA, Magee L, Monteiro CA, Saxena S, Millett C. Sugar and artificially sweetened beverage consumption and adiposity changes: National longitudinal study. *International Journal of Behavioral Nutrition and Physical Activity.* 2015;12.
4. Lobstein T, Baur L, Uauy R, TaskForce IIO. Obesity in children and young people: a crisis in public health. *Obesity Reviews*2004;5 Suppl 1:4-104.
5. Must A, Barish EE, Bandini LG. Modifiable risk factors in relation to changes in BMI and fatness: what have we learned from prospective studies of school-aged children? *Int. J. Obes.* 2009;33(7):705-715.
6. Brophy S, Cooksey R, Gravenor MB, et al. Risk factors for childhood obesity at age 5: Analysis of the Millennium Cohort Study. *BMC Public Health.* 2009;9.
7. Morenga LT, Mallard S, Mann J. Dietary sugars and body weight: systematic review and meta-analyses of randomised controlled trials and cohort studies. *BMJ,* 2013;346.
8. Massion S, Wickham, S., Pearce, A., Barr, B., Law, C., & Taylor-Robinson, D. Exploring the impact of early life factors on inequalities in risk of overweight in UK children: findings from the UK Millennium Cohort Study. *Arch. Dis. Child.* 2016.
9. Scottish Centre for Social Research. Growing Up in Scotland Sweep 5 - 2009/10 Data Documentation Scottish Centre for Social Research nd.

10. Bradshaw P, Marryat, L., Mabelis, J., Ferrandon, M. Tipping, S. . Growing up in Scotland Sweep 5: 2009-2010 User Guide Scottish Centre for Social Research nd.
11. Scottish Government. Revised Dietary Goals for Scotland Scottish Government 2014.
12. Parkes A, Sweeting, H., Wight, D. *Growing Up in Scotland Overweight, obesity and activity.* Edinburgh: Scottish Government 2012.
13. Scottish Government. Physical activity guidelines for early years (under 5s) - for children who are capable of walking; Scottish Government 2011.
14. Scottish Government. Physical activity guidelines for children and young people (5-18 years) Scottish Government; 2011.
15. Bradshaw P, Marryat, L., Corbett, J., Ferrada, M. . Growing Up in Scotland Sweep 4: 2008 - 2009 User Guide. Scottish Centre for Social Research nd.
